# Detection of Integrons in *Escherichia coli* Associated With Urinary Tract Infections in Riyadh, Saudi Arabia

**DOI:** 10.1155/ijm/1013136

**Published:** 2025-10-13

**Authors:** Enshad Alzaidi, Maryam Alshammari, Manal AlKhulaifi, Bader Alrashidi, Abdulkarim Alhetheel, Reem Aljaaidi, Dunia Al Farraj

**Affiliations:** ^1^Department of Botany and Microbiology, King Saud University, Riyadh, Saudi Arabia; ^2^College of Applied Medical Sciences, Inaya Medical Colleges, Riyadh, Saudi Arabia; ^3^Department of Pathology, College of Medicine, King Saud University, Riyadh, Saudi Arabia

**Keywords:** antibiotic resistance, biofilm formation, Class 1 integron, Class 2 integron, Class 3 integron, *Escherichia coli*, urinary tract infections

## Abstract

Treatment of *Escherichia coli* infections has become increasingly challenging due to the emergence of multidrug-resistant mechanisms within the bacterial genome. Integrons play an essential role in spreading antibiotic resistance. This study is aimed at detecting the prevalence of Class 1, 2, and 3 integrons among *E. coli* associated with urinary tract infections (UTIs). A total of 90 *E. coli* strains were isolated from UTI samples and tested for antibiotic susceptibility using phenotypic methods. Biofilm formation was conducted using the microtiter plate method. Conventional PCR was used to detect the integrase genes. Overall, 48.9% of *E. coli* isolates (44/90) were MDR, and 58.9% (53/90) were resistant to ampicillin. A total of 60% (54/90) of *E. coli* isolates were biofilm producers. PCR results showed that 22.2% (20/90), 6.7% (6/90), and 3.3% (3/90) of *E. coli* isolates were positive for Class 1, Class 2, and both classes of integrons, respectively. However, Class 3 integron was not detected in all *E. coli* isolates. A significant correlation was observed between the MDR and Class 1 integron (*p* < 0.05). There is no statistical significance between the presence of integrons and biofilm formation among *E. coli* isolates. Our findings revealed that the presence of Class 1 integron among *E. coli* isolates was associated with antibiotic resistance.

## 1. Introduction

**Figure 1:**
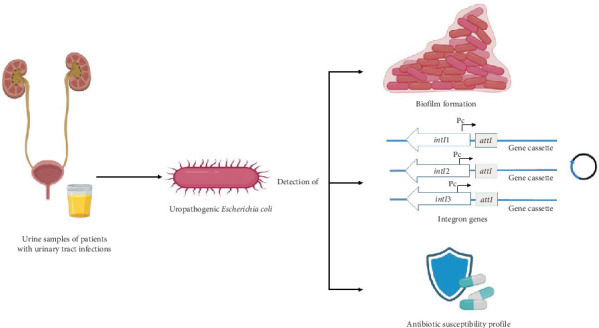
Schematic representation of a proposed approach to detect the relation between integron genes, antibiotic resistance, and biofilm formation. The figure was created with BioRender (https://biorender.com/).

Urinary tract infections (UTIs) are one of the most common bacterial infections, and they are becoming more common in both community and clinical settings [1]. Despite attempts to improve UTI diagnostic and treatment options, *E. coli* strains are prevalent among UTIs and represent the majority of community-acquired infections (95%) and hospital-acquired infections (50%) [2, 3]. Bacterial antibiotic resistance is a leading problem throughout the world, and the treatment of uropathogenic *Escherichia coli* (UPEC) has become more challenging and highly complicated because of several drug resistance mechanisms to existing antimicrobials [4–9]. Among all, the major route of resistance gene transmission is via mobile genetic elements (MGEs), including insertion sequences (ISs), transposons (Tns), plasmids, and integrons (Ints) [10, 11]. Ints are MGEs typically containing one gene cassette (GC) or more than one, which are present in the bacterial genome within Tns, chromosomes, and plasmids. *E. coli* carries Ints within the bacterial genome, which is a major threat due to the expression and spreading of genes responsible for antibiotic resistance [2, 12]. Structurally, Int comprises three essential parts: (1) integrase gene (*intI*), (2) recombination site (*attI*), and (3) promoter (Pc) [12]. To date, approximately 130 various cassettes conferring antibiotic resistance with special *attC* sites have been identified [13, 14]. More than nine Int classes were characterized based on the amino acid sequences of integrase genes, and four classes were identified in clinical bacterial isolates. Ints are found more in Gram-negative bacteria than in Gram-positive bacteria. Nevertheless, Classes 1 to 3 are dominant in the multidrug resistance (MDR) of Gram-negative bacteria, including *E. coli*, which is related to unsuccessful antibiotic treatment, but Classes 1 and 2 are the most common among clinical isolates [12, 14–16]. In the 1980s, approximately 3% of *E. coli* isolates were associated with Class 1 Ints, but by the 2010s, that number had increased to 26% [17]. Analysis of Int genes is crucial to determine drug-resistant patterns among *E. coli* strains in the human population. Limited information is available about the distribution of Ints among *E. coli* isolates isolated from UTIs in Saudi Arabia. Thus, the present study is aimed at evaluating the prevalence of Class 1, 2, and 3 Ints among *E. coli* strains isolated from UTI cases in Riyadh City (Figure 1).

## 2. Materials and Methods

### 2.1. Sample Collection and Bacterial Isolates

A total of 90 *E. coli* were isolated from urine samples of patients with UTIs at a bacteriology laboratory at King Saud University Medical City (KSUMC). Urine samples were inoculated on MacConkey agar. The culture plates were incubated for 24 h at 37°C. *E. coli* isolates were identified using the VITEK MS system (bioMérieux, France) (VITEK2 GN card) and the MicroScan WalkAway 96 Plus System (Siemens, Tarrytown, New York) (NBC50).

### 2.2. Antibiotic Susceptibility Testing

Susceptibility profiles to various antibiotics were performed using the VITEK MS system (VITEK 2 AST-N291 card; bioMérieux, Inc., Durham, North Carolina, United States) and the MicroScan WalkAway 96 Plus System. All *E. coli* isolates were tested using prescribed guidelines [18]. Both systems provide results for antibiotics as resistant (R), intermediate (I), and sensitive (S). If *E. coli* strains showed drug resistance to at least one antibiotic from three or more different antibiotic classes, they were recognized as MDR [19]. To determine antibiotic susceptibility and virulence factor analysis, two MTCC *E. coli* strains (ATCC 35218 and ATCC 25922) were used for analysis. These control bacterial strains were used for all experiments.

### 2.3. Detection of Biofilm Formation

Biofilm formation was conducted as previously described by Kord et al. with some modifications [20]. Bacterial suspensions were prepared in 3 mL LB broth according to 0.5 MacFarland. Then, 200 *μ*L of each inoculum was added to 96-well flat-bottom microtiter plates (Nest Biotechnology, China) and incubated overnight at 37°C. The plates were further washed to remove planktonic cells. Then, the developed biofilms were stained using crystal violet for 8 min (225 *μ*L; 1%). The microtiter plate was washed with double-distilled water. After the plates were air-dried, acetic acid (225 *μ*L) was added and mixed. Then, the absorbance was read at 560 nm (OD_560_) by a multiplate reader (Synergy 2, BioTek). The final results were categorized as strong biofilm formation, moderate biofilm formation, weak biofilm formation, and negative for biofilm formation (Table 1). Isolates with weak, moderate, and strong biofilm formation were referred to as biofilm producers. The positive control strain, *E. coli* ATCC 25922, was used as a biofilm-forming producer. LB broth media was used as the negative control.

### 2.4. Genomic DNA Extraction

A pure colony of *E. coli* isolates that had grown on nutrient agar (NA) plates was added to 500 *μ*L of nuclease-free water. Then, 400 *μ*L of the suspension was used for DNA extraction using an automated system (EZ1 Advanced XL, Qiagen) with a DNA purification kit (EZ1 Virus Mini Kit v2.0, Qiagen, Germany) as per the manufacturer's instructions. Then, the purified DNA was kept at −80°C for Int gene detection.

### 2.5. Molecular Detection of Class 1, 2, and 3 Int Genes

Amplification of Int genes *intI1*, *intI2*, and *intI3* from *E. coli* isolates was performed by the conventional polymerase chain reaction (PCR) method (GeneAmp PCR System 9700, Applied Biosystems) using specific primers (Table 2). PCR analysis was performed with the final volume of 20 *μ*L containing 2X AmpMaster Taq (10 *μ*L) (GeneAll Biotechnology Co., Ltd.), forward primer (1 *μ*L), reverse primer (1 *μ*L), purified DNA template (1 *μ*L), and nuclease-free water (7 *μ*L). Negative control was prepared in each run by adding a PCR mixture without the DNA template. The thermal cycling conditions of the PCR reaction were done in this study, as previously described, with some modifications [22]. The program for *intI1* was set as follows: initial denaturation (2 min, 95°C), followed by denaturation (30 cycles) for 1 min at 94°C, annealing for 1 min at 56°C, extension (72°C, 1 min), and final extension (72°C, 10 min). The *intI2* and *intI3* programs were similar to those of *intI1*, but the cycle number was 25, and the annealing temperatures were 59°C and 64°C, respectively. PCR amplicons were loaded in submarine gel electrophoresis in 1.2% (*w*/*v*) agarose gel (Bioline) stained with DyeAll (GeneAll Biotechnology Co., Ltd.). The separated DNA was visualized and documented using the Gel Doc XR+ Imaging System (Bio-Rad Laboratories, Inc.). GENESTA 100 bp DNA Ladder (GeneAll Biotechnology Co., Ltd.) was used to determine the size of the amplicons.

### 2.6. Statistical Analysis

Statistical analysis was performed using SPSS software (Version 22). The chi-square test was used to analyze the relation between the Int genes, antibiotic resistance, and biofilm formation among *E. coli* isolates. *p* < 0.05 was considered statistically significant.

## 3. Results

### 3.1. Bacterial Isolates and Antibiotic Susceptibility Profiles

In the present study, a total of 90 *E. coli* were isolated and selected for antibiotic susceptibility profiles. Among UTI patients, 88.9% (80/90) of the *E. coli* strains were obtained from females, and the remaining 11.1% (10/90) were from males. The range of age was 4 weeks to 90 years, and the average age was 42.4 years (Table 3). The susceptibility patterns of 14 selected antibiotics for all 90 isolated *E. coli* strains are presented in Table 4. About 58.9% (53/90) of the *E. coli* isolates were resistant to ampicillin, as it showed the maximum resistance percentage, followed by 36.7% (33/90) to trimethoprim/sulfamethoxazole. The isolates were much more sensitive to tigecycline (100%). Moreover, 44 (48.9%) of the screened *E. coli* isolates were MDR, which are resistant to at least one antibiotic among three or more antibiotic groups, while 22 of these isolates produced ESBL (24.4%).

### 3.2. Detection of Biofilm Formation Among *E. coli* Isolates

*E. coli* isolates were examined for biofilm formation using the microtiter plate assay method. In general, 60% (54/90) of the bacterial strains have the potential to produce biofilm, while 40% (36/90) were considered as non-biofilm producers (negative) (Table 5). The present study revealed that both biofilm-producing and non-biofilm-producing organisms showed antibiotic resistance. Accordingly, the biofilm formation mechanism did not affect antibiotic resistance, ESBL production, and MDR (*p* > 0.05) (Table 4).

### 3.3. Occurrence of Class 1, 2, and 3 Int Genes in *E. coli* Strains

**Figure 2:**
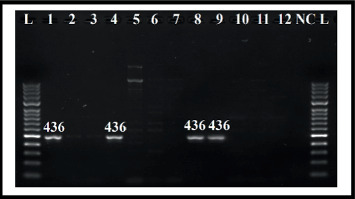
Detection of amplified *intI1* genes from *E. coli* strains using agarose gel electrophoresis. Note. Lane *L* = 100 bp DNA marker; Lane (1–12) = amplified *intI1* gene; Lane (1, 4, 8, and 9) = *E. coli* amplicon positive for *intI1*; lane NC = negative control.

**Figure 3:**
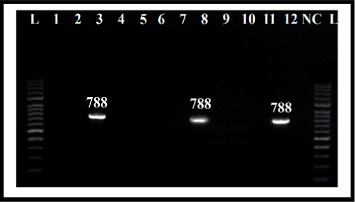
Detection of amplified *intI2* genes from *E. coli* strains using agarose gel electrophoresis. Note. Lane *L* = 100 bp DNA marker; Lane (1–12) = amplified *intI2* gene; Lane (3, 8, and 12) = *E. coli* amplicon positive for *intI2*; lane NC = negative control.

The present study was focused on the three classes of Ints (*intI1*, *intI2*, and *intI3*), which may be directly related to antibiotic resistance using conventional PCR. Out of 90, 20 isolates (22.2%) carried *intI1*, and six isolates (6.7%) were positive for *intI2*. However, *intI3* was not identified in all isolates. Moreover, three of the isolates (3.3%) were positive simultaneously for *intI1* and *intI2* genes (Figures 2 and 3). The Class 1 Int was associated with antibiotic (ciprofloxacin, ampicillin, and trimethoprim/sulfamethoxazole) resistance and was statistically significant (*p* < 0.05). *E. coli* with Class 2 Int gene showed association with antibiotic resistance to gentamicin, ampicillin, piperacillin/tazobactam, and amoxicillin/clavulanic acid (*p* < 0.05). Also, the simultaneous presence of both Class 1 and 2 Ints was associated with drug resistance to gentamicin, piperacillin/tazobactam, amoxicillin/clavulanic acid, and trimethoprim/sulfamethoxazole (*p* < 0.05) (Table 4). The presence of both Classes 1 and 2 together had a weak correlation with ESBL production (*p* = 0.08). A significant correlation was observed between the MDR and only Class 1 Int (*p* value < 0.001) (Table 4).

The ability of *E. coli* isolates containing Class 1 and 2 Ints to produce biofilms is presented in Table 6. A total of 65% (13/20), 66.7% (4/6), and 33.3% (1/3) of *E. coli* isolates contained Class 1 Int, Class 2 Int, and both Class 1 and 2 Ints, which can form biofilms. There is no statistical significance between the presence of Ints and the development of biofilms (*p* > 0.05).

## 4. Discussion

The prevalence of Int classes among bacterial cells as MGEs is one of the major contributing factors to the spreading of antibiotic resistance [23]. In the Kingdom of Saudi Arabia, *E. coli* is considered one of the major prevalent bacteria, contributing to 42.2% of infections, as its infection rate increased during the period from 2015 to 2019 by 10.2% [24]. The present study analyzes the Int classes and their frequency among pathogenic *E. coli* isolated from UTI patients in Riyadh City.

Recently, *E. coli* were identified as a major contributor to UTIs and show a major threat to human health [25]. The majority of UPEC bacteria analyzed in this study were resistant to ampicillin (58.9%), followed by trimethoprim/sulfamethoxazole (36.7%), as none of the isolates were found to be resistant to tigecycline. The present findings were consistent with previous reports in Saudi Arabia, and the detected *E. coli* strains were highly resistant to ampicillin (66.3%), followed by trimethoprim/sulfamethoxazole (48.5%) [9]. Recently, Bazaid et al. reported that all UPEC isolates were susceptible to ampicillin, but 29% were resistant to tigecycline, contradicting the present study [26]. In addition, our findings showed that 24.4% of the isolated *E. coli* strains produced ESBL. According to previous studies, 75%, 59%, 33.49%, and 15.7% of *E. coli* strains isolated from UTI patients in Iran, Morocco, Saudi Arabia, and the United States synthesized ESBL [27–30].

Our findings also found that 48.9% of UPEC strains were highly resistant to more than three antibiotic classes. In 2018, the MDR rate in *E. coli* characterized from UTIs in Saudi Arabia was 22.77%, which is much lower than the present findings [9]. The rate of MDR in recent studies conducted in Iran, Iraq, and Jordan was 93.6%, 69.3%, and 57.3%, respectively, which is higher than the present findings [31–33]. However, a study performed in six European countries revealed that the MDR rate varied from 1.7% to 26.9% [34].

Biofilm formation in *E. coli* contributes to prolonging its survival in the urinary system and thus increases the difficulty, severity, and recurrence of UTIs [31].

Additionally, 60% of the *E. coli* isolates exhibited biofilm formation, with similar rates in Uganda, Nepal, and India [35–37]. Another study in Iran revealed a higher rate of biofilm formation (99%) [31]. The present finding revealed that antibiotic resistance was not associated with biofilm formation, and non-biofilm-forming bacteria also have drug resistance. The biofilm-forming *E. coli* strains in this study were more resistant to tested drugs than non-biofilm-forming strains, except gentamicin, ciprofloxacin, trimethoprim/sulfamethoxazole, cefepime, and ceftazidime. However, biofilm-producing bacteria did not show a significant relationship with antibiotic resistance. Other findings revealed that biofilm formation by *E. coli* protects them from antibiotic treatment. Unlike planktonic bacteria, biofilm-forming bacteria may be up to 1000 times more resistant [38–40]. Similarly, the close presence of bacterial cells within biofilms may make it easier for the transformation of virulence genes [37]. Neupane et al. indicated that high concentrations of antibiotics are necessary to prevent the growth of biofilm-forming bacteria [41]. In this study, the rate of MDR isolates that formed biofilms was 51.9%, which was statistically nonsignificant when compared to isolates that did not form biofilms. This finding is inconsistent with studies that revealed a positive correlation between biofilm development and MDR in *E. coli* strains isolated from urine samples [35, 40].

The genes carried by Ints are purposed to provide antibiotic resistance mechanisms, as Class 1, 2, and 3 Ints in Enterobacteriaceae are associated with the spread of genes responsible for drug resistance [42]. Class 1 Ints were highly distributed among *E. coli* clinical isolates (22.2%) compared to Classes 2 and 3 in our research, consistent with previous literature [22, 43, 44]. It has been reported that Class 1 Ints have been detected in 8.75% to 70% of clinical *E. coli* isolates [2, 22, 43–46]. In this study, Class 2 Ints were found in 6.7% of the strains, which is comparable with the previous report in Iran, Azerbaijan, and Iraq, but a few other reports in Iran reported the higher emergence of Class 2 Ints by 14.1% and 21% [22, 43–45, 47, 48]. Alkhudhairy et al. found both *intI1* and *intI2* simultaneously in 3.3% of *E. coli* isolated from UTIs, as in this study, while 8.5% of these classes were reported by Yekani et al. [44, 47]. PCR amplification and analysis revealed the absence of Class 3 Ints, and this result was consistent with the majority of previous reports [2, 22, 42, 44]. Moreover, the prevalence of Class 3 Int in 6.5% of *E. coli* strains from different clinical sources in Saudi Arabia has been reported [15].

The present study observed a significant correlation between Class 1 Ints and ciprofloxacin, ampicillin, and trimethoprim/sulfamethoxazole resistance, while Class 2 Ints were associated with gentamicin, ampicillin, amoxicillin/clavulanic acid, and piperacillin/tazobactam resistance (*p* < 0.05). The present findings revealed that the copresence of Class 1 and 2 Ints in *E. coli* strains significantly correlated with gentamicin, amoxicillin/clavulanic acid, piperacillin/tazobactam, and trimethoprim/sulfamethoxazole resistance (*p* < 0.05). The present findings revealed that antibiotic resistance is more common in *E. coli* isolates carrying Class 1 Ints than in isolates lacking this class of genes. In agreement with the present results, the presence of Class 1 Ints was associated with resistance to ampicillin, ciprofloxacin, nalidixic acid, and trimethoprim/sulfamethoxazole as reported by Mirnezami et al.; to imipenem, ceftazidime, streptomycin, and trimethoprim/sulfamethoxazole as reported by Akya et al.; and to tobramycin and lower resistance to aztreonam as reported by Chen et al. [42, 43, 49]. Interestingly, Class 1 Int genes significantly encoded drug-resistant mechanisms in *E. coli* compared to other classes [45]. In contrast, the clinical Class 2 Int gene is a silent gene that restricts the potential of Ints to acquire and reorganize GCs [50, 51]. Yekani et al. reported that Class 2 Ints are dysfunctional because of the early in-frame stop codon in the bacteria [44]. In addition, the lack of statistical significance between drug resistance and the presence of Int classes or the presence of antibiotic resistance in isolates that are negative for Int genes can be explained by the fact that these isolates can acquire resistance through other MGEs or enzymes encoded in the chromosomes [43, 52]. Despite the absence of Class 3 Int genes in our investigation, the prevalence of this class of Ints that carry a range of GCs expressing MDR is a severe concern on a global scale [53]. Additionally, the current study showed that 31.8% of the isolated ESBL-secreting *E. coli* strains showed Int genes. There was an insignificant correlation between ESBL production and the presence of any Int classes (*p* > 0.05). Moghaddam et al. revealed that almost all ESBL-producing *E. coli* characterized from UTIs in Iran were coded with only Class 1 Ints [54]. In a study performed in Syria, Class 1 Ints were reported in 73.9% of the ESBL-producing UPEC strains, and there was a significant correlation between them [46].

MDR and Ints are highly correlated, particularly in Enterobacteriaceae, including *E. coli* [22]. The study demonstrated that MDR in *E. coli* was strongly linked to Class 1 and 2 Ints (85% and 83.3%), with a significant correlation between MDR and the presence of Class 1 Int (*p* < 0.001) and a weak correlation between MDR and Class 2 Int (*p* = 0.081). The high dissemination of Class 1 and 2 Ints in MDR *E. coli* isolates indicates that these elements provide a selection advantage to bacterial strains as increased use of antibiotics leads to selective pressures in hospital settings [55]. Previous studies indicated a significant correlation between the existence of Ints in clinically isolated *E. coli* and MDR [15, 22, 44, 45]. In this study, all isolated *E. coli* strains that exhibited both Class 1 and 2 Ints simultaneously were 100% MDR and statistically nonsignificant (*p* = 0.072).

GCs for Ints are transported and captured more easily in biofilms [56]. In the present study, the presence of Int genes among biofilm-forming *E. coli* isolates was investigated. It found a negative correlation between the biofilm-producing property and Int classes (*p* > 0.05). Class 1 Int and biofilm formation in UPEC are significantly correlated and were reported previously in Iran [39]. There have been insufficient studies to conclude the relationship between Int genes and biofilm-forming properties of bacterial strains isolated from UTIs in Saudi Arabia. A study found high rates of Int genes in biofilm-forming *P. aeruginosa* isolated from the hospital environment and clinical subjects, specifically Class 1 and 2 Ints [57].

The present findings may help establish appropriate programs in disinfection strategies to control and prevent UTIs and reduce biofilm formation and drug resistance.

## 5. Limitations

One limitation of this study is the lack of consideration for patient-related factors, such as other illnesses or previous antibiotic use, which may influence the dissemination of Int genes and the expression of biofilm formation genes.

## 6. Conclusion

To our knowledge, this is the first report in Riyadh City, Saudi Arabia, that discussed the prevalence of Int classes in *E. coli* associated with UTIs and the relation between Int genes, antibiotic resistance, and biofilm formation. *E. coli* associated with UTIs were more resistant to ampicillin compared to other antibiotics, as the resistance rate exceeded 50%. Also, 60% of *E. coli* isolates had the ability to form a biofilm. Our findings revealed the prevalence of Ints, especially Classes 1 and 2, among *E. coli* isolates, while Class 3 was not detected. Class 1 and 2 Ints showed a close association with resistance to certain antibiotics. Remarkably, *E. coli* isolates that carried both Class 1 and 2 Ints had a weak relation with ESBL production. Additionally, *E. coli* isolates that carried Class 1 Ints had significantly higher levels of MDR. It is important to analyze other MGEs that are involved in drug resistance in *E. coli* to develop an appropriate treatment strategy.

## Figures and Tables

**Table 1:** Biofilm formation categories.

**Biofilm formation capacity**	**Mean of optical density**
Negative	OD ≤ ODc
Weak	2 × ODc ≥ OD > ODc
Moderate	4 × ODc ≥ OD > 2 × ODc
Strong	OD > 4 × ODc

*Note:*ODc = average OD of negative control + (standard deviations [SD] of negative control × 3).

Abbreviation: ODc = optical density cutoff.

**Table 2:** Specific primers for the detection of integrase genes in *E. coli.*

**Integrase gene**	**Primer sequence**	**Size**	**Reference**
*intI1*	F: GGTCAAGGATCTGGATTTCG	436 bp	[21]
	R: ACATGCGTGTAAATCATCGTC		
*intI2*	F: CACGGATATGCGACAAAAAGG	788 bp	
	R: TGTAGCAAACGAGTGACGAAATG		
*intI3*	F: AGTGGGTGGCGAATGAGTG	600 bp	
	R: TGTTCTTGTATCGGCAGGTG		

**Table 3:** Frequency of *E. coli* strains among ages and genders.

**Gender**	**Age group (%)**				**Total**
	**≤18**	**19–40**	**41–60**	**≥61**	
Females	13 (14.4)	31 (34.4)	12 (13.3)	24 (26.7)	80 (88.9)
Males	2 (2.2)	2 (2.2)	2 (2.2)	4 (4.4)	10 (11.1)
Total	15 (16.7)	33 (36.7)	14 (15.6)	28 (31.1)	90

**Table 4:** Antibiotics susceptibility profiles and their relationship to the presence of integrons and biofilm formation in *E. coli* isolates.

**Antibiotic**	**(+) *intI1* (** **n** = 20**)**		**(+) *intI2* (** **n** = 6**)**		**(+) *intI1* and *intI2* (** **n** = 3**)**^**a**^		**BF (** **n** = 54**)**		**Total** **R** **(** **n** = 90**) (%)**
	**R** **(%)**	**p** ^**b**^	**R** **(%)**	**p** ^**b**^	**R** **(%)**	**p** ^**b**^	**R** **(%)**	**p** ^**b**^	
AN	0	0.44	0	0.7	0	0.79	2 (3.7)	0.24	2 (2.2)
GM	3 (15)	0.09	2 (33.3)	0.007^c^	2 (66.7)	<0.001^c^	3 (5.6)	0.61	6 (6.7)
CIP	10 (50)	0.03^c^	1 (16.7)	0.46	1 (33.3)	0.9	16 (29.6)	0.93	27 (30)
AM	18 (90)	0.001^c^	6 (100)	0.03^c^	3 (100)	0.14	32 (59.3)	0.93	53 (58.9)
AMC	3 (15)	0.71	3 (50)	0.03^c^	2 (66.7)	0.02^c^	12 (22.2)	0.18	16 (17.8)
TZP	1 (5)	0.34	1 (16.7)	0.01^c^	1 (33.3)	<0.001^c^	2 (3.7)	0.24	2 (2.2)
SXT	19 (95)	<0.001^c^	4 (66.7)	0.11	3 (100)	0.02^c^	18 (33.3)	0.42	33 (36.7)
FEP	6 (30)	0.7	3 (50)	0.18	2 (66.7)	0.11	12 (22.2)	0.24	24 (26.7)
FOX	3 (15)	0.4	1 (16.7)	0.57	1 (33.3)	0.17	6 (11.1)	0.67	9 (10)
CAZ	7 (35)	0.49	3 (50)	0.24	2 (66.7)	0.14	14 (25.9)	0.45	26 (28.9)
TGC	0	—	0	—	0	—	0	—	0
FM	2 (10)	0.5	0	0.5	0	0.64	4 (7.4)	0.73	6 (6.7)
IPM	1 (5)	0.34	0	0.7	0	0.79	2 (3.7)	0.24	2 (2.2)
MEM	0	0.59	0	0.79	0	0.85	1 (1.9)	0.41	1 (1.1)
ESBL	6 (30)	0.51	3 (50)	0.13	2 (66.7)	0.08	10 (18.5)	0.11	22 (24.4)
MDR	17 (85)	<0.001^c^	5 (83.3)	0.08	3 (100)	0.07	28 (51.9)	0.49	44 (48.9)

Abbreviations: (+), positive for integron gene; AM, ampicillin; AMC, amoxicillin/clavulanic acid; AN, amikacin; BF, biofilm formation (weak, moderate, and strong); CAZ, ceftazidime; CIP, ciprofloxacin; FEP, cefepime; FM, nitrofurantoin; FOX, cefoxitin; GM, gentamicin; IPM, imipenem; MEM, meropenem; R, resistant and intermediate; SXT, trimethoprim/sulfamethoxazole; TGC, tigecycline; TZP, piperacillin/tazobactam.

^a^The presence of both Class 1 and 2 integrons in a single isolate.

^b^Pearson's chi-square test.

^c^Statistically significant.

**Table 5:** Degrees of biofilm formation among *E. coli* strains.

**Biofilm-forming ability**	**No. of isolates (%)**
Negative (not form biofilm)^a^	36 (40)
Weak^b^	39 (43.3)
Moderate^c^	8 (8.9)
Strong^d^	7 (7.8)
Total	90

*Note:* Biofilm-forming ability was classified as weak, moderate, and strong. The negative control was defined by a cutoff (ODc = 0.08).

^a^OD ≤ ODc (0.08).

^b^2 × ODc (0.164) ≥ OD > ODc.

^c^4 × ODc (0.328) ≥ OD > 2 × ODc (0.164).

^d^OD > 4 × ODc (0.328).

**Table 6:** Analysis of biofilm formation among *E. coli* strains harboring integron classes.

**Integron class**	**No. of *E. coli* isolates (%)**		**Total**	**p** **value** ^**a**^
	**Biofilm-forming**	**Non-biofilm-forming**		
*intI1*	13 (65)	7 (35)	20	(0.605)
*intI2*	4 (66.7)	2 (33.3)	6	(0.73)
*intI1* and *intI2*^b^	1 (33.3)	2 (66.7)	3	(0.338)

*Note:* Biofilm-forming = weak + moderate + strong.

^a^Pearson's chi-square test.

^b^Both Class 1 and 2 integrons were concurrently harbored in a single *E. coli* isolate.

## Data Availability

The data that support the findings of this study are available from the corresponding author upon reasonable request.
